# ASSESSMENT OF A MICROPLATE SYSTEM FOR MEASURING INDIVIDUAL REAL-TIME RESPIRATION IN SMALL MODEL ORGANISMS OF AGING

**DOI:** 10.1093/geroni/igz038.3347

**Published:** 2019-11-08

**Authors:** Ashley N Turner, Jessica M Hoffman, Mickie L Powell, Melissa J Sammy, Douglas R Moellering, Tim R Nagy, Steven N Austad, Daniel L Smith

**Affiliations:** 1 Department of Biology, University of Alabama at Birmingham, Birmingham, Alabama, United States; 2 Department of Nutrition Sciences, University of Alabama at Birmingham, Birmingham, Alabama, United States

## Abstract

The ability to measure oxygen consumption rates of a living organism in real-time provides an indirect method of monitoring dynamic changes in metabolism reflecting organismal level mitochondrial function. In this study, we assessed the Loligo Systems microplate system for measuring individual respiration in small organisms. This included adult nematodes (Caenorhabditis elegans, N2), zebrafish embryos (Danio rerio, AB), and adult fruit flies (Drosophila melanogaster, w1118). Organisms were placed inside 80 µL glass chambers on a 24-well microplate atop a 24-channel optical fluorescence oxygen reading device. Adult nematodes and zebrafish embryos were in liquid culture, M9 buffer and egg water respectively, and the adult flies were in room air. The microplate and reader were placed inside an incubator for temperature control. A silicone gasket with a thin liner was used to seal the chambers. Reference standard oxygen consumption (respiration) of single and multiple adult nematodes (n=1–4 animals/well), zebrafish embryos (n=1–4 animals/well), and adult flies (n=1–2 animals/well) in the microplate system were achieved. Significant differences across numbers of animals/well and by sex were observed. Validation experiments of the oxygen consumption rates measured in C. elegans in parallel with Seahorse extracellular flux (XF) experiments are underway. The Loligo Systems microplate system offers a non-invasive, non-destructive method to measure real-time respiration in smaller organisms. These data provide preliminary evidence for utility of the system for a variety of biomedical applications that relate to organismal and mitochondrial function/dysfunction, including research in the basic biology of aging in these highly-utilized, pre-clinical, genetic model organisms.

